# Cytokine Kinetics during Progression of COVID-19 in Rwanda Patients: Could IL-9/IFNγ Ratio Predict Disease Severity?

**DOI:** 10.3390/ijms241512272

**Published:** 2023-07-31

**Authors:** Ella Larissa Ndoricyimpaye, Jacques Van Snick, Rutayisire Robert, Emmanuel Bikorimana, Onesphore Majyambere, Enatha Mukantwari, Thaddée Nshimiyimana, Valens Mbonigaba, Jean Paul Coutelier, Nadine Rujeni

**Affiliations:** 1 Department of Biomedical Laboratory Sciences, School of Health Sciences, College of Medicine and Health Sciences, University of Rwanda, Kigali P.O. Box 3248, Rwanda; ella.ndoricyimpaye@uclouvain.be (E.L.N.); robertrutayisire@gmail.com (R.R.); monesphore@gmail.com (O.M.); nshimiyimanath@gmail.com (T.N.); jean-paul.coutelier@uclouvain.be (J.P.C.); 2 de Duve Institute, Université Catholique de Louvain, 1348 Brussels, Belgium; 3 Ludwig Institute for Cancer Research, Universite Catholique de Louvain, 1348 Brussels, Belgium; jacques.vansnick@uclouvain.be; 4 National Reference Laboratory, Rwanda Biomedical Center, Kigali P.O. Box 4285, Rwanda; ntwarie@gmail.com (E.M.); mbova2020@gmail.com (V.M.); 5 Department of General Nursing, School of Nursing, College of Medicine and Health Science, University of Rwanda, Kigali P.O. Box 3248, Rwanda; emmanuelbikori@gmail.com

**Keywords:** SARS-CoV-2, biomarkers, immune response, IL-9, IFNγ

## Abstract

For effective treatments and preventive measures against severe COVID-19, it is essential to determine early markers of disease severity in different populations. We analysed the cytokine kinetics of 129 COVID-19 patients with mild symptoms, 68 severe cases, and 20 healthy controls for the first time in Rwanda. Pro-inflammatory (IFNγ, IL-6, TNFα), Treg (IL-10, TGFβ1, TGFβ3), Th9 (IL-9), Th17 (IL-17), and Th2 (IL-4, IL-13) cytokines, total IgM and IgG, as well as gene expressions of *FoxP3, STAT5+, IFNγ-R1*, and *ROR alpha+,* were measured at day 1, day 7, day 14, day 21, and day 28 post-infection. Severe cases showed a significantly stronger increase than mild patients in levels of all cytokines (except IL-9) and all gene expression on day 1 of infection. Some cytokine levels dropped to levels comparable to mild cases at later time points. Further analysis identified IFNγ as a marker of severity throughout the disease course, while TGFβ1, IL-6, and IL-17 were markers of severity only at an early phase. Importantly, this study revealed a striking low IL-9 level and high IFNγ/IL-9 ratio in the plasma of patients who later died compared to mild and severe cases who recovered, suggesting that this could be an important biomarker for predicting the severity of COVID-19 and post-COVID-19 syndrome.

## 1. Introduction

The clinical manifestations of COVID-19, a viral infection due to SARS-CoV-2, vary from individual to individual and even between communities with different genetic backgrounds [1]. In particular, there is a wealth of evidence suggesting that COVID-19 was less severe in Africa compared to other continents, possibly due to demography, social economy, genetics, and a ‘trained immunity’ [2,3,4,5]. However, little is known about immune correlates among individuals with different clinical manifestations of COVID-19. Moreover, there is currently limited evidence on using biologics in COVID-19 to prevent severe illness or improve survival [6].

COVID-19 clinical manifestations range from fever to multiple organ failure through dry cough and pneumonia [7,8]. The ‘cytokine storm’, characterised by excessive secretion and release of high levels of cytokines by a dysfunctional immune system, has been associated with severe COVID-19 [9]. This systemic hyper-inflammation is closely associated with ARDS and/or multiple organ damage, which ultimately can lead to death [10].

Innate and adaptive immune responses to SARS-CoV-2 are heterogeneous. For instance, IL-6 and IL-8 have been associated with diagnostic and COVID-19 severity [11]), while the expression of IFNγ was reduced in severely ill patients [12]. On the other hand, it has been demonstrated that Th1 and Th17 cells can induce an inflammatory response in patients with COVID-19, leading to a high risk of death [13,14]. Regulatory cells were also moderately increased in severe patients, suggesting immunosuppression in COVID-19 [13]. Interestingly, individuals exposed to other coronaviruses before the COVID-19 pandemic may have developed antibodies cross-reacting with SARS-CoV-2, protecting them from severe disease [2,12]. In animal models, Th2 cytokines (IL-13) were associated with mortality [14]. Although it is evident that immune responses play a significant role in COVID-19 clinical outcomes, the mechanisms underlying this heterogeneity are still elusive [15]. It is possible that genetic diversity can explain these variations [1], but the mechanistic characterisation of immune responses will help identify early markers of disease severity in different settings and populations.

Cytokine profiles have been suggested as potential biomarkers for viral infections such as influenza or MERS [16]. Antibodies have also been associated with COVID-19 outcomes [6]. To provide timely interventions for COVID-19 and prevent death, a comprehensive understanding of the cytokine and antibody kinetics during disease progression is needed. This study aimed to assess a wide range of cytokines and T cell transcription factors to determine the most discriminating cytokine kinetics in disease severity and death outcomes in patients with COVID-19 in Rwanda. We hypothesized that severe disease could be predicted in this setting based on immune markers.

## 2. Results

### 2.1. Patients Characteristics

A total of 197 patients (mild = 129, severe = 68) with COVID-19 and 20 healthy controls were included in this study. Patients’ baseline characteristics, treatments, and outcomes are shown in Table 1. Severe disease was characterised by fever, dyspnea, hypoxia, oxygen saturation, difficulty breathing, and multi-organ dysfunction, while mild disease was defined by fever and/or cough. Patients with severe COVID-19 were older than those with mild clinical manifestations (Kruskal–Wallis test, *p* < 0.0001).

### 2.2. Kinetic Analysis of Cytokine and Antibody Levels in the Plasma of COVID-19 Patients

**Figure 1:**
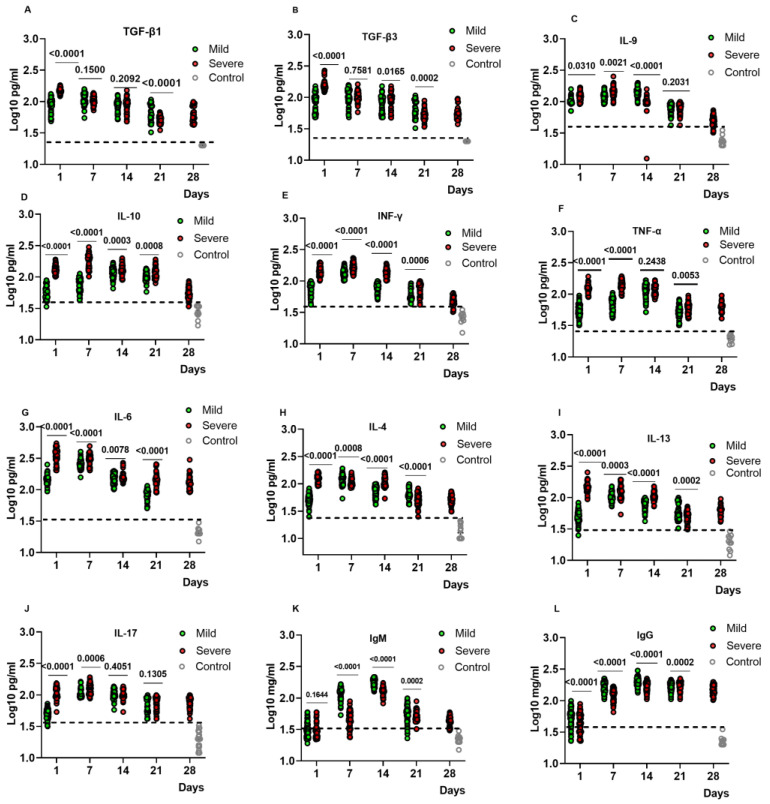
Cytokine and immunoglobulin levels in COVID-19 patients and healthy controls. Kinetic analysis of the plasma levels (pg/mL) for cytokines: TGF-β1 (**A**), TGF-β3 (**B**), IL-9 (**C**), IL-10 (**D**), IFNγ (**E**), TNFα (**F**), IL-6 (**G**), IL-4 (**H**), IL-13 (**I**), IL-17 (**J**) and for immunoglobulins levels (mg/mL): IgM (**K**), IgG (**L**) at different time points. Mean plasma concentrations of cytokines and total immunoglobulins were compared between severe and mild groups using one-way ANOVA. The dotted lines show the limit of healthy control levels.

We analysed the kinetic changes of inflammatory and regulatory cytokine levels, including IL-4, IL-6, IL-9, IL-10, IL-13, IL-17, TNFα, IFNγ, TGFβ1, and TGFβ3 (active forms). The changes in total IgG and IgM were also analysed. All cytokine plasma levels were increased in infected patients compared to controls, with significant fluctuation of cytokine levels in mild and severe patients (Figure 1). For most cytokines, including IFNγ, TNFα, IL-4, IL-6, IL-10, IL-13, and IL-17, the concentrations were higher in severe than mild cases at all time points. This difference was evident at the earliest time points, likely due to a higher viral load in patients that later developed more severe diseases. In contrast, TGF-β1 and TGF-β3, although higher in severe cases on day 1 after infection, were similarly increased in both groups on days 7 and 14, and, on day 21, the mild group even showed the highest levels (Figure 1A,B). A similar difference was seen for IL-9 levels that were always equivalent or slightly higher in mild patients than in the severe group (Figure 1C).

IgG and IgM were increased in both severe and mild patients until day 14, followed by a decrease in IgM in both groups of patients. IgG sustained its increase during the disease course (Figure 1L,M). We further calculated the ratio IgM/IgG and found a higher ratio in severe compared to mild cases in the early phases (Supplementary S1).

### 2.3. Gene Expressions of FoxP3, STAT5+, IFNγ-R1, and ROR Alpha+ of COVID-19 Patients

**Figure 2:**
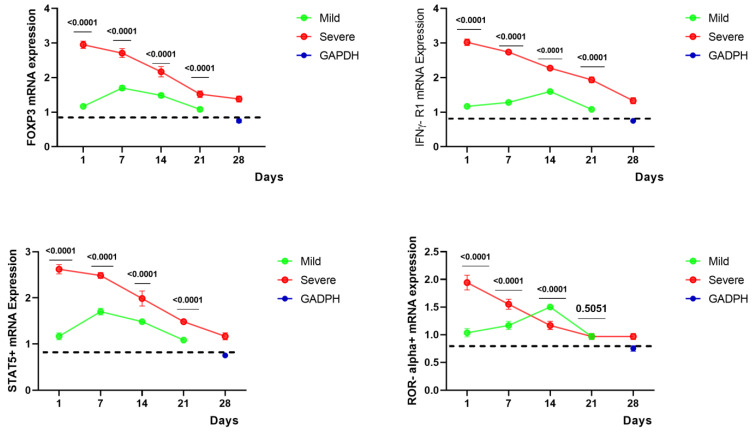
*FoxP3, STAT5+, IFNγ-R1, and ROR alpha+* Gene expression. RNA was extracted from the blood drawn from SARS-CoV-2-infected patients and healthy donors. Kinetic gene expression analysis of different T cell populations in COVID-19 patients was analysed at different times. The cycle threshold (ΔCT of gene expression) was calculated against the *GAPDH* (internal control). Mean ± SEM was plotted, and mixed ANOVA was used to calculate the group differences. The dotted lines show the limit of internal control (*GAPDH*).

More robust upregulation of mRNA expression for *FoxP3, STAT5+, ROR alpha+,* and *IFNγ R1* was detected in severe COVID-19 patients compared to mild groups. Indeed, a statistically significant upregulation of *FoxP3, IFNγ-R1* (3-fold changes), and *STAT5+* (2.5-fold changes) were observed in severe COVID-19 patients compared to mild groups (*p* < 0.0001) on day one of disease detection. Subsequently, a continuous downregulation of *FoxP3* and *IFNγ R1* mRNA followed in severe and mild cases. On the other hand, *ROR alpha*+mRNA showed a significant upregulation of around two-fold in severe as compared to mild patients (*p* < 0.0001) in the early days of disease onset, followed by a reverse at later stages (Figure 2).

### 2.4. Prognostic Factors for the Identification of Severe COVID-19 Cases

**Figure 3:**
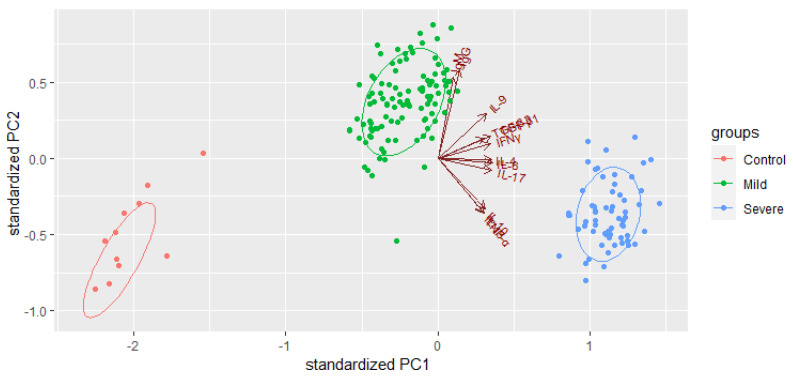
Prognostic factors of severe COVID-19. The R package “factoextra” performed principal component analysis to identify correlated variables for distinguishing severe and mild COVID-19 patients.

**Figure 4:**
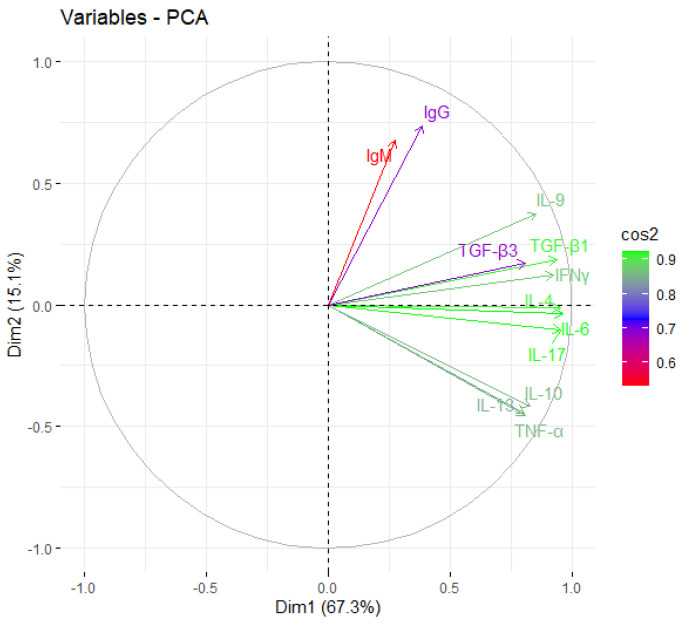
Principal component analysis was performed by R package “factoextra”—the most contributing variable to severe COVID-19 patients. The most contributing variables are in green, with the least contributing factor in red.

**Figure 5:**
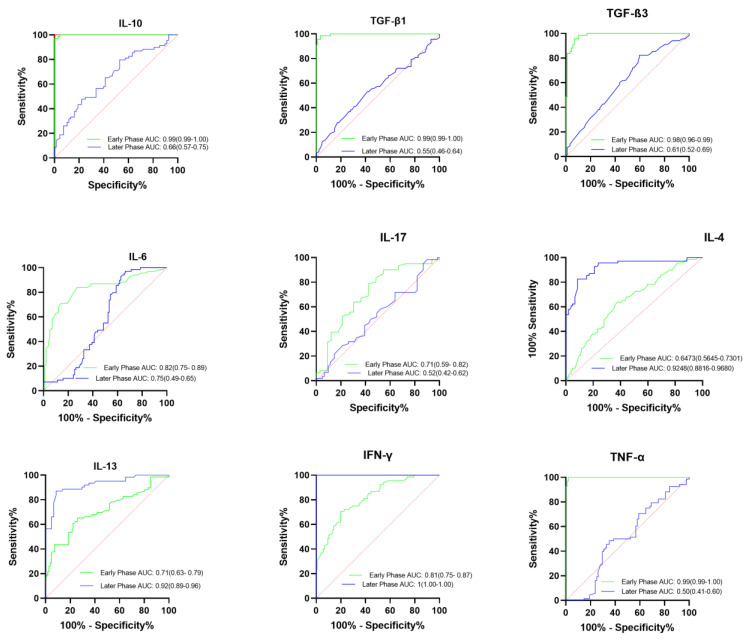
Biomarker for COVID-19 severity. The receiver-operating characteristic (ROC) analysis with the area under the receiver-operating curve (AUC) ranging from 0 to 1 discriminating severe from mild COVID-19 patients in the early and late phases when age and sex were adjusted as confounding factors.

Principal Component Analysis (PCA) was first performed using the R package “factor extra” to identify correlated variables for distinguishing severe groups from mild and control groups. Using the cytokine profiles of COVID-19 patients, we assessed the possible prognostic markers of the severity of the disease. Th2 cytokines (IL-4, IL-13), regulatory cytokines (IL-10, TGF-β1, TGF-β3), Th-17 cytokines (IL-17), and pro-inflammatory cytokines (IL-6, TNFα, IFNγ), were skewed towards severe cases (Figure 3). The most contributing variables, IL-6, IL-4, IL-17, IFNγ, and TGF-β1, were further analysed statistically (Figure 4). Using Prism GraphPad software, the receiver-operating characteristic (ROC) curve and the area under the ROC curve (AUC) were calculated to assess the diagnostic value of the most contributing cytokines in the early and late phases of the disease. The results of this analysis identified IFNγ as the disease severity factor with the highest value for both the early (AUC: 0.82) and late (AUC = 1.00) phases of the disease. TGF β1 was the best biomarker of severity during the early phase of infection (AUC: 0.99), followed by IL-10 (AUC: 0.99), IL-6 (AUC: 0.82), and IL-17 (AUC: 0.71). At the same time, IL-4 (AUC: 0.92) and IL-13 (AUC: 0.71) were identified as markers of severity at a later phase (Figure 5).

### 2.5. Comparison of Cytokine Profiles of Patients with Severe COVID-19 and the Deceased

**Figure 6:**
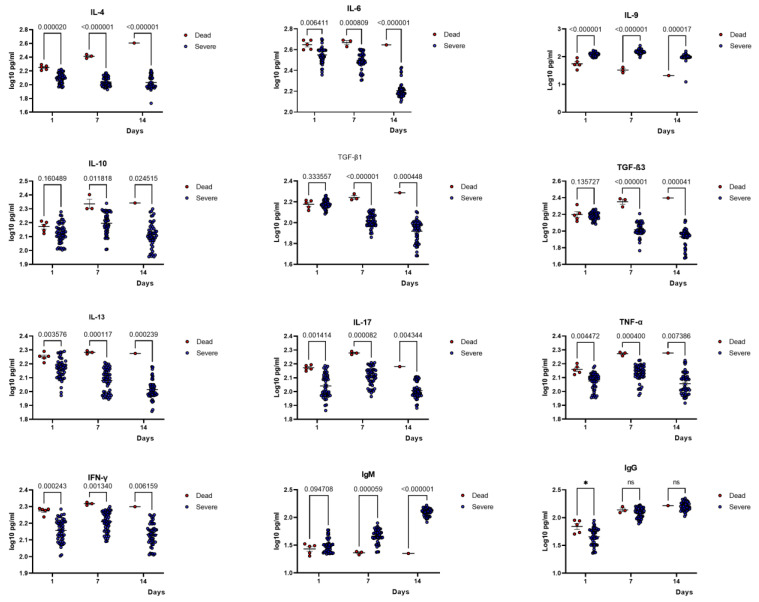
Comparison of cytokines concentration between recovered patients and patients who later died. Kinetic analysis of cytokines (pg/mL) and immunoglobulins concentrations of COVID-19 patients who later died compared with those who survived at three different time points after hospital admission. Statistics: Wilcoxon T-test was used to compare mean cytokine/antibody concentrations between groups.

To further narrow our analysis for identifying immune markers of disease severity, we divided the severe patients into two groups: patients who later died from COVID-19 and those who recovered. Remarkably, patients who died had higher plasma concentrations of IL-4, IL-6, IL-10, IL-13, IL-17, TGF-β1, TGF-β3, IFNγ, and TNF-α throughout the 14 days (when they were still alive) compared to severe survivor patients. On the contrary, IL-9 plasma concentrations were low at the baseline in patients who later died compared to those who recovered, followed by a significant decrease on days 7 and 14 with *p* < 0.0001 and *p* < 0.0001, respectively (Figure 6). IL-9 levels remained high in survivors.

### 2.6. The Ratio IFNγ/IL-9 and Disease Severity/Death

Given the importance of IFNγ in our predictive analysis and the association between IL-9 and survival, we analysed the kinetics of IFNγ/IL-9 ratio at the onset of infection. Comparison between mild and severe cases at baseline (day 1) indicates a significantly high ratio in the severe group (*p* = 0.003) (Table 2).

## 3. Discussion

COVID-19 remains a public health threat, and assessing determinants for severe disease is highly interesting. Using a wide range of cytokine profiles of COVID-19 patients, we evaluated the possible prognostic parameters for predicting the severity of the disease. We found that all cytokines analysed, with the exception of IL-9, were increased from the earliest time point in severe patients compared to healthy controls. Moreover, severe cases showed higher cytokine levels at the earliest time, probably reflecting a higher viral load in patients who later developed the more aggressive disease. This aligns with the proposed ‘cytokine storm’ in COVID-19′s severe form [17].

Contrary to other cytokines, IL-9 plasma levels were significantly lower in patients who died later than in surviving individuals. This is probably the result of the inhibition of Th9 cells by IRF-1 [18], which SARS-CoV-2 strongly upregulates due to the massive production of Type I, II, and III interferons during coronavirus infection [19]. This aligns with the high IFNγ levels observed in our severely infected patients and the higher IFNγIL-9 ratio in patients who later died of infection, making this IFNγ/IL-9 ratio a novel marker of disease severity. As reported previously, IFNγ can negatively regulate IL-9-producing Th9 and Th17 cell differentiation in vitro and in vivo through the induction of IL-27 [20]. A protective role of IL-9 in infectious immunopathology has been reported earlier. For example, IL-9 has been shown to contribute to controlling *Trypanosoma cruzi* infections in Chagas disease’s immunopathology by neutralising the inflammatory response and increased fibrosis while limiting parasitemia [21]. High levels of IL-9 through TGF-β induction were also negatively associated with severe malaria [22].

Apart from the predictive power of the IFNγ/IL-9 ratio, this study’s findings indicate that severe patients with COVID-19 expressed high levels of all tested cytokines. This included pro-inflammatory cytokines, such as TNFα, IL-6, and IFNγ, the latter being the most striking immunological marker of severe disease. IFNγ is an antiviral cytokine expressed by Th1 T cells, NK cells, and C8^+^Tcells [23]. The increase of IFNγ indicates a Th1-skewed response during COVID-19, and the increase in mRNA IFNγR1 transcription factor further supported this. Our findings on pro-inflammatory cytokines align with a meta-analysis of 23 studies indicating that high levels of pro-inflammatory cytokines such as IL-6 and TNFα are associated with severe disease [24]; however, Th2 cytokines (IL-4 and IL-13) were also upregulated in severe/dying patients. Th2 cytokines are predominantly associated with fibrogenic inflammatory remodelling by inducing the alternative activation of macrophages (M2) and the release of TGFβ by fibroblast and platelet-derived factors [25]. Moreover, the overproduction of Th2 cytokines can result in chronic inflammation, as in leishmaniasis, where the activation of IL-4 and IL-13 results in pathogen expansion and chronic infection [25,26].

This study’s high levels of anti-inflammatory cytokines were associated with COVID-19 severity and mortality, in line with previous reports [13]. The high levels of IL-10 may be interpreted as an attempt to temper hyperinflammation and prevent tissue damage. However, other studies demonstrated that the concurrent elevations of IL-10 and other various inflammatory cytokines suggest the failure of IL-10 to suppress inflammation [27] or act as an immunostimulatory molecule [28].

TGFβ is a potent immunoregulatory cytokine. Indeed, our study on malaria patients suggested that TGFβ could prevent severe disease [22]. Others have demonstrated that SARS-CoV-2 uses TGFβ to dampen the immune response, allowing viral persistence [29], and an increase in blood TGFβ during severe COVID-19 may be associated with disseminated intravascular coagulation (contained in alpha granules of platelets) [30]. In line with these studies, we found higher TGF-β1 and TGF-β3 in the plasma of patients with severe COVID-19 compared to patients with mild disease at the onset of infection, the trend reversing at later stages of infection [15,31]. These findings suggest that TGFβ may have failed to modulate severity at early stages, possibly due to the ‘cytokine storm’ [32]. One possible reason for this failure could be that our patient’s TGFβ, which is a potent inducer of IL-9 [33], failed to stimulate IL-9 production, probably because of the inflammatory context with a high IL-6/IL-4 balance and instead stimulated IL-17, which may have contributed to disease severity in COVID-19 patients. Indeed, it has been reported that high serum levels of IL-17 and other cytokines are associated with severe COVID-19 [34,35]. Furthermore, IL-17 was linked with persistent viraemia by inhibiting the apoptosis of infected cells in synergy with IL-6 [36]. Consistently, significantly higher IL-17 and IL-6 were found in severely ill patients in our cohort. In addition, a weak positive correlation between IL-17 and TGFβ1 and TGFβ3 was noted in the early phase of the disease, although IL-17 was not the best predictor of disease severity.

Given that asymptomatic/mild COVID-19 could progress to severe disease, identifying early biomarkers that could timely predict the severity risk is a promising strategy to prevent morbidity and mortality due to SARS-CoV-2. Previous studies have highlighted the use of biomarkers as predictors for disease severity during viral infections, such as Epstein–Bar virus [37], cytomegalovirus infection [38], hepatitis C virus [39], as well as influenza virus [40]. The current study identifies IFNγ as the strongest predictor of severity (persisting throughout the disease in severely ill patients). In addition, TGFβ1, TGFβ3, IL-10, and IL-6 were prognostic makers for the early phase of COVID-19 severity, consistent with previous reports. Indeed, TGF-β1 levels on day 7 exhibited high sensitivity and specificity to distinguish COVID-19 patients with lung damage from those without [41]. In addition, IL-10 and IL-6 had higher odds of progression to severe phenotype in a meta-analysis of 6320 patients [42].

Studies have associated COVID-19 pathology with T cell differentiation pathways [43,44]. Our findings highlight the role of T cell differentiation-related genes in the outcomes of COVID-19. In our cohort, *FoxP3, IFNγ-R1*, and *STAT5+* gene expressions of T cell populations were significantly upregulated in severe COVID-19 compared to mild patients. This was in line with the increased cytokine levels observed in severe cases of COVID-19 since *FoxP3, IFNγ-R1*, and *STAT5+* mRNA promotor genes are involved in T cell differentiation towards Treg, Th1, Th2, and Th17, and their corresponding cytokine milieu. Consistent with these findings, previous studies have reported that the upregulation of *FoxP3* coincided with the development of severe hypoxia and the death of patients [45]. However, Stephen-Victor et al. suggested that the levels of functional T regs are reduced as the severity of COVID-19 increases [46]. Similarly, a recent study has reported that severe COVID-19 was related to elevated levels of IL-2 induced through IL-2R-JAK-*STAT5* signalling pathways [12].

The levels of mRNA *ROR alpha+,* a transcription factor for Th17 cells, were higher in severe cases than in mild, but the reverse was true on day 14, and they were comparable later on. Studies have suggested a role for the ROR gene in disease severity and lung damage [47,48]. However, it has been demonstrated that IL-9 can induce Th17 differentiation and enhance functional nTregs [49]. This is in line with the role of functional Tregs in reducing COVID-19 severity [46] and the observed increase in IL-9 among patients who recovered from severe disease in our cohort.

Our study has limitations; we could not assess the effect of antiviral treatment and immunomodulating agents due to the changes in treatment protocol over the recruitment period. Furthermore, we could not determine the exact time of the first clinical manifestation as all the study participants were enrolled from day 1 of the positive COVID-19 test. However, we uncovered significantly different cytokines in mild and severe COVID-19. All severely ill patients were admitted to the hospital for the severity of their illness rather than isolation.

In conclusion, our study confirmed that pro-inflammatory cytokines are associated with severe COVID-19. In particular, IFNγ and IFNγ/IL-9 ratios are powerful biomarkers of COVID-19 severity and may predict the potential for survival. In addition, TGFβ1, TGFβ3, IL-6, and IL-10 may be influential biomarkers of COVID-19 severity during the early phase of infection, whereas Th2 cytokines, IL-4 and IL-13, may be markers of persisting severity.

## 4. Materials and Methods

### 4.1. Study Population

A prospective observational study was conducted in Rwanda on COVID-19 patients admitted at the Musanze COVID-19 treatment centre and home-based patients attending healthcare facilities in Kigali. Patients were enrolled on the day of hospital admission (severe cases) and at first presentation (and confirmation of infection) in health care testing centres (mild cases). Blood samples were taken for cytokine measurement and qRT-PCR for molecular biomarkers. Patients were followed up for 28 days with 7-day intervals between the blood collection time points (i.e., day 1, day 7, day 14, day 21, and day 28, depending on when patients cleared infection or died).

### 4.2. Severity of COVID-19

The severity of COVID-19 was classified as mild and severe, as described previously [8]. Mild cases were defined as light COVID-19 clinical symptoms only without pneumonia. Severe cases confirmed pneumonia, respiratory distress, respiratory rate ≥30/min, oxygen saturation ≤ 93% in room air, patients who needed ventilation, developed shock, or had other organ failures requiring admission to critical care.

### 4.3. Testing SARS-CoV-2

The viral load of each patient was determined by taking nasopharyngeal swabs for synthesized patients (severe) for a real-time PCR, as described [50]. The home-based patients were tested using SARS-CoV-2 antigenic tests [51].

### 4.4. Cytokine Profile Using ELISA

Blood samples were collected into EDTA tubes after a SARS-CoV-2 positive test was confirmed with follow-up time points of blood collection. Blood was immediately cooled on ice for laboratory processing. Plasma was separated by centrifugation (2000× *g* for 15 min) and stored in 300 microliter aliquots at −80 °C until analysis. The plasma concentration of 10 cytokines (IL-4, IL-6, IL-10, IL-13, IL-17, TNFα, IFNγ) and two immunoglobulins (IgG, IgM) was measured upon first thaw using a quantitative enzyme-linked immunosorbent assay (ELISA). ELISA was performed according to the manufacturer’s protocol (Wuhan Fine Biotechnology Ltd., Wuhan, China). As previously described in our study, TGF-β1,3 (active forms) and IL-9 cytokines were analysed using homemade ELISA reagents [22]. All cytokine (ng/mL) and immunoglobulin levels (mg/mL) were measured in duplicate), and each plate included a standard curve and negative control.

### 4.5. Gene Expression Evaluation

Isolation of total RNA from the whole blood with a Rneasy Mini Kit (Qiagen, Hilden Germany) was performed according to the manufacturer’s instructions. cDNA was ynthesized as previously described [22]. The amount of gene expression of *FoxP3, STAT5A, RORAlpha+, and IFNγ-R1 was* detected using qRT- PCR on ABI 7500 real-time PCR system (Applied Biosystems, Waltham, MA, USA). Primer probes of *FoxP3* (5′-AACTATGAAACAAATTTTCCT3′ and 5′-TTAGGAAAATTTGTTTCATAG-3′), *STAT5+* (5′-GTTCAGTGTTGGCAGCAATGAGC-3′ and 5′-ACCACCCTGTTGCTGTACCAA-3′), *ROR alpha+*(5′-CACCAGCATCAGGCTTCTTTCC-3′ and 5′-GTATTGGCAGGTTTCCAGATGCG-3′) and *IFNγ-R1* (5′-GAGTGTGGAGACCATCAAGGAAG-3′ and 5′-TGCTTTGCGTTGGACATTCAAGTC-3′) from OriGene Technologies (Rockville, USA) were used to analyse for different gene expressions against *GADPH* (5′-GTCTCCTCGATTCAACAGCG-3′ and 5′-ACCACCCTGTTGCTGTACCAA-3′) as an internal control. The programme was set up following a constant protocol: the first step at 50 °C for 2 min, initial heating at 90 °C for 10 min, 50 cycles of denaturation at 95 °C for 15 s, annealing at 60 °C for 1 min, and elongation at 60 °C for 1 min. The samples were assayed in duplicate, and the average cycle threshold (ΔCT of gene expression) was calculated, while GAPDH was used as the internal control housekeeping gene. The relative fold expression changes were determined.

### 4.6. Statistical Analysis

One-way ANOVA and chi-squared test were used to assess the differences between continuous and categorical clinical variables across three groups of COVID-19 patients. Continuous data were described with the median, while categorical variables were represented as proportions. Differences were considered statistically significant for the p-value less than 0.05. Principal component analysis using the Factor extra R package was used to determine the most contributing cytokines to the severity of the disease, with age and sex as confounding variables. Pearson correlation was used to assess the association between cytokines. The ratio of pro-inflammatory cytokines to anti-inflammatory cytokines was calculated. Receiver-operating characteristic (ROC) curve analysis using GraphPad Prism 9.4.1 (San Diago, CA, USA) was applied to determine the most discriminating biomarkers between patients with severe and mild disease at different stages of disease progression. Age and sex as confounding factors were controlled to calculate the area under the ROC (AUC) value. All ELISA data were log-transformed.

## Figures and Tables

**Table 1:** Patient characteristics, treatment, and outcome.

	Control (*n* = 20)	Mild (*n* = 129)	Severe (*n* = 68)	* p-Value
Age (years-median (IQR))	34 (23–44)	34 (26–44)	42 (31–70)	<0.0001
Gender (F/M)	14/6	47/82	42/26	-
Presence of comorbidities	0 (0)	21 (16.28)	9 (13.43)	0.5996
Oxygen Therapy-no. (%)	0 (0)	0	68 (100)	<0.0001
Mechanical Ventilation-no. (%)	0 (0)	0 (0)	53 (78)	<0.0001
Analgesic and antipyretic-no. (%)	0 (0)	97 (75)	10 (15)	<0.0001
Steroids-no. (%)	0 (0)	30 (23)	59 (87)	0.065
Remdesivir-no. (%)	0 (0)	25 (19)	60 (88)	0.0636
Antibiotics-no. (%)	0 (0)	80 (62)	40 (59)	0.050
Hospital Mortality-no. (%)	0 (0)	0 (0)	5 (7.46)	0.004

n: the number of participants. * Kruskal–Wallis for continuous variable and chi-squared for categorical variables tests were used for statistical significance level for comparing mild and severe groups. All values are expressed in the median and interquartile range unless specified.

**Table 2:** Ratio of IFNγ over IL-9 between severe and mild groups.

Variables	Mean	Std. Deviation	Coefficient of Dispersion	Coefficient of Variation	T-Test Ratio
				Mean Centered	
Control	0.842	0.064	0.028	6.8%	
Mild	0.832	0.053	0.045	5.7%	
Severe	1.002	0.045	0.033	4.5%	T = 8.595, *p* = 0.003
Overall	0.957	0.061	0.050	6.4%	

The ratio of IFNγ over IL-9 between severe and mild cases at day one (baseline) time points.

## Data Availability

The datasets presented in the current study are available from the corresponding authors upon reasonable request.

## Supplementary Materials

The following supporting information can be downloaded at: https://www.mdpi.com/article/10.3390/ijms241512272/s1.
