# Supplementary figures and images for: Cytokine Kinetics during Progression of COVID-19 in Rwanda Patients: Could IL-9/IFNγ Ratio Predict Disease Severity?
Source: Int J Mol Sci. 2023 Jul 31;24(15):12272. doi: 10.3390/ijms241512272 (PMC10418469; doi:10.3390/ijms241512272)

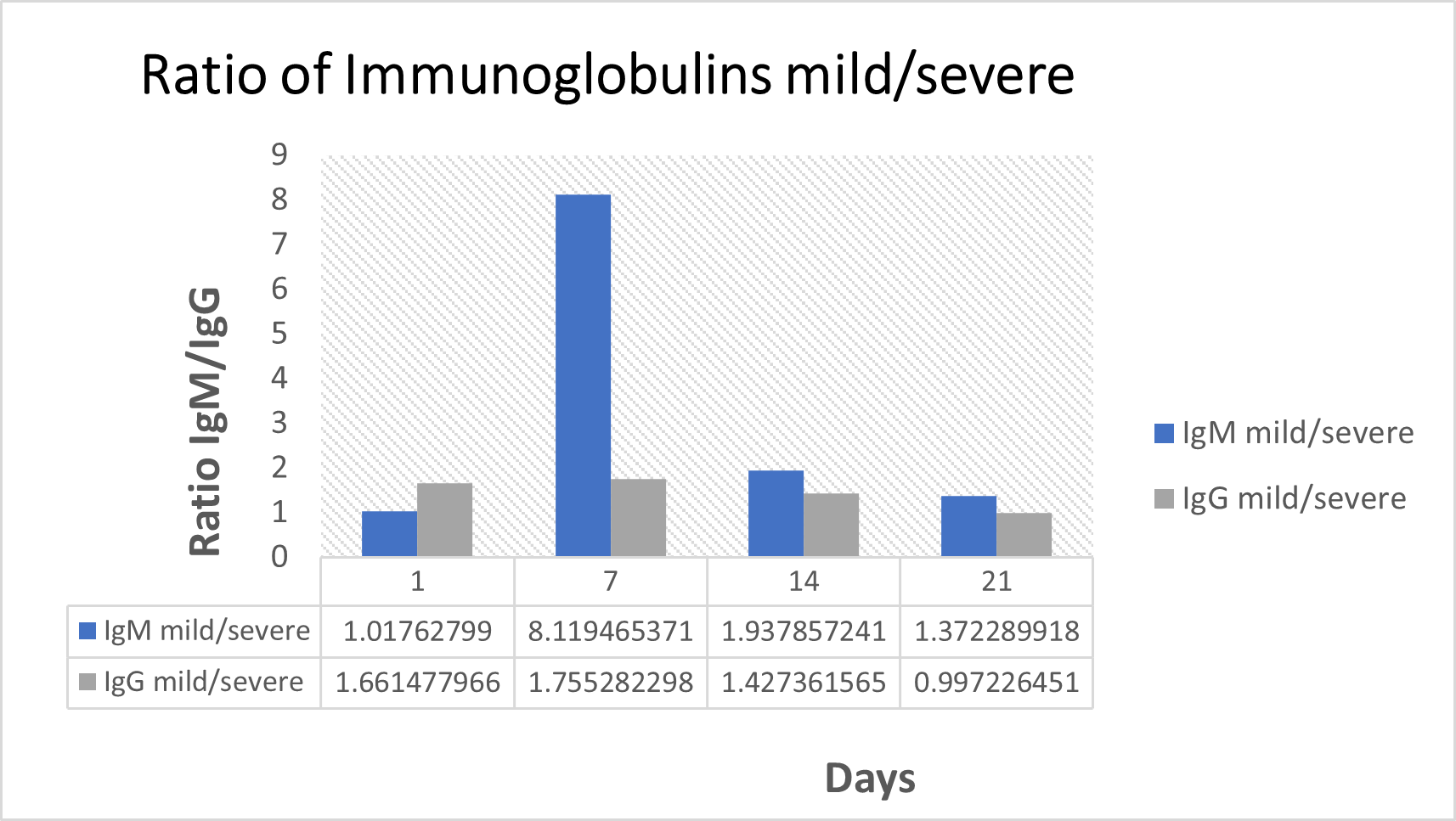

Supplement: Supplementary file 1 [file ijms-24-12272-s001.zip › ijms-2412921-SI.tif]
